# Supplementary material for: Simultaneous Quantitation of S(+)- and R(−)-Baclofen and Its Metabolite in Human Plasma and Cerebrospinal Fluid using LC–APCI–MS/MS: An Application for Clinical Studies
Source: Molecules. 2020 Jan 8;25(2):250. doi: 10.3390/molecules25020250 (PMC7024189; doi:10.3390/molecules25020250)

Supplementary Materials

Simultaneous Quantitation of S(+)- and R(−)-Baclofen and Its Metabolite in Human Plasma and Cerebrospinal Fluid using LC–APCI–MS/MS: An Application for Clinical Studies

Qingfeng He [1,†], Yashpal S. Chhonker [1,†], Matthew J. McLaughlin [2], and Daryl J. Murry [1,3,*]

[1] Clinical Pharmacology Laboratory, Department of Pharmacy Practice and Science, University of Nebraska Medical Center, Omaha, NE 68198, USA; qingfeng.he@unmc.edu (Q.H.); y.chhonker@unmc.edu (Y.S.C.)

[2] Division of Rehabilitation Medicine, Children's Mercy Kansas City, UMKC School of Medicine, Kansas City, MO 64108, USA; mjmclaughlin@cmh.edu

[3] Fred and Pamela Buffett Cancer Center, University of Nebraska Medical Center, Omaha, NE 68198, USA

* Correspondence: dj.murry@unmc.edu; Tel.: +1-402-559-3790 or 402-559-2430

† Authors contributed equally to this manuscript

Simultaneous quantitation of R- and S-CHBA metabolite and racemic baclofen in human plasma and cerebrospinal fluid using

Liquid chromatographic and mass spectrometric (LC/MS) conditions

R- and S-CHBA and racemic baclofen assay: Chromatographic separations were performed using a Chiralcel OJ R-RH column (2.1mm x150 mm, 5 μ, Daicel Chemical Industries, Ltd., Japan) along with a C18 guard column (EVO-C18; 2.1 mm internal diameter, Phenomenex, Torrance, CA.). The system was operated in isocratic mode with a mobile phase composed of 0.4% formic acid in water and 0.4% FA in ACN (84:16, v/v) operated at a flow rate of 1.0 mL/min. The chromatographic separation was achieved using a 27 minute total run time. A 10 μL injection volume was adopted for all samples.

Post-column infusion of 0.5% ammonium hydroxide (NH_4OH) in water and ACN (80:20, v/v) operated at 0.1 mL/min was performed using a 3-way connector after the column and prior to mass detection.

The MS parameters were optimized using the auto-optimization method as implemented in the LabSolutions software Version 5.6 (Shimadzu Scientific, Inc, Columbia, MD, USA). The final parameters for each analyte and IS are shown in **Table 1**. LabSolutions software Version 5.6 was used to perform data acquisition and quantitation.

Preparation of stock, calibration standards and quality control samples

Stock solutions of R- and S- baclofen, R-CHBA, and S-CHBA were prepared by dissolving 1 mg of each compound into 1 mL water. The stock solutions were transferred to an eppendorf vial and stored at -20°C for future use.

For quantitation of total racemic baclofen and individual R- and S-CHBA isomers, the same CCs and QCs were reinjected on the metabolite separation method. Concentrations of CCs ranged from 1 to 2000 ng/mL for R- and S- CHBA and from 2 to 4000 ng/mL for racemic baclofen. QCs for racemic baclofen include 2 ng/mL LLOQ, 10 ng/mL of LQC, 1000 ng/mL of MQC and 3000 ng/mL of HQC. The corresponding concentrations for R- and S-CHBA were half that for the total racemic baclofen. The QCs concentration and assay performance are shown in Table 3. All the samples were kept at -20 °C until analysis.

For the internal standard, 1 mg/mL stock solution of Baclofen-d4 (racemic) was prepared by dissolving 1 mg in 1 mL water. The sample was transferred to an eppendorf vial and stored at -20°C for future use. When preparing the working IS stock solution, the baclofen d4 was diluted with 50% methanol to make a 5 μg/mL solution.

Assay Validation

The developed LC-MS/MS assay for R- and S-CHBA separation was validated for sensitivity, selectivity, accuracy and precision in plasma according to the current FDA guidelines [25].

Using a Chiralcel column to separate R- and S-CHBA metabolites and total baclofen, the %RSD of inter-day precision values for QC samples were between 3.3 to 18.7% with intra-day precision values ranging from 1.7 to 9.7%, which indicated acceptable assay precision **(Supplementary Table 1)**.

Supplementary Table 1. Intra and inter-day precision (% RSD) and accuracy (% bias) for baclofen and its metabolites in human plasma.

**Conc. (ng/mL)**	**Baclofen *(racemic)***				***S*-CHBA**				***R-CHBA***			
	**LLOQ**	**LQC**	**MQC**	**HQC**	**LLOQ**	**LQC**	**MQC**	**HQC**	**LLOQ**	**LQC**	**MQC**	**HQC**
Theoretical Conc.	2 ng/mL	10 ng/mL	1000 ng/mL	3000 ng/mL	1 ng/mL	5 ng/mL	500 ng/mL	1500 ng/mL	1 ng/mL	5 ng/mL	500 ng/mL	1500 ng/mL
$\%Bias_{intra\text{-}assay}$	−4.8	3.1	−1.8	−1.3	5.9	−0.7	−7.4	−5.3	0.2	−6.2	−13.7	−8.9
$\%Bias_{inter\text{-}assay}$	−3.7	12.0	−3.6	−2.0	9.7	−4.2	−7.1	−12.4	12.2	−1.1	−7.4	−11.7
$\%\ RSD_{intra\text{-}assay}$	6.3	8.4	1.0	4.6	7.6	7.5	8.2	1.7	3.3	9.7	4.4	5.6
$\%\ RSD_{inter\text{-}assay}$	9.7	8.5	7.2	8.5	16.3	5.9	12.7	10.5	18.7	10.6	14.9	4.6

Supplementary Figure 1. Representative MRM ion-chromatograms of (a) blank plasma using the conditions for racemic baclofen showing interfering peak at 4.5 min, (b) plasma spiked with baclofen (rt, 5.0 min., 10 ng/mL), (c) extracted patient sample, (d) blank plasma using the conditions for CHBA metabolite, (e) plasma spiked with CHBA metabolite showing the two isomers (S-CHBA 21.0 min and R-CHBA 23.5 min, at 5 ng/mL), (f) extracted patient sample showing only the S-CHBA metabolite is present in plasma, (g) blank plasma using the conditions for baclofen-d4 showing interfering peak at 4.5 min, (h) plasma spiked with IS (baclofen-d4; rt, 5.0 min., 1000ng/mL), (i) extracted patient sample spiked with IS (baclofen-d4).

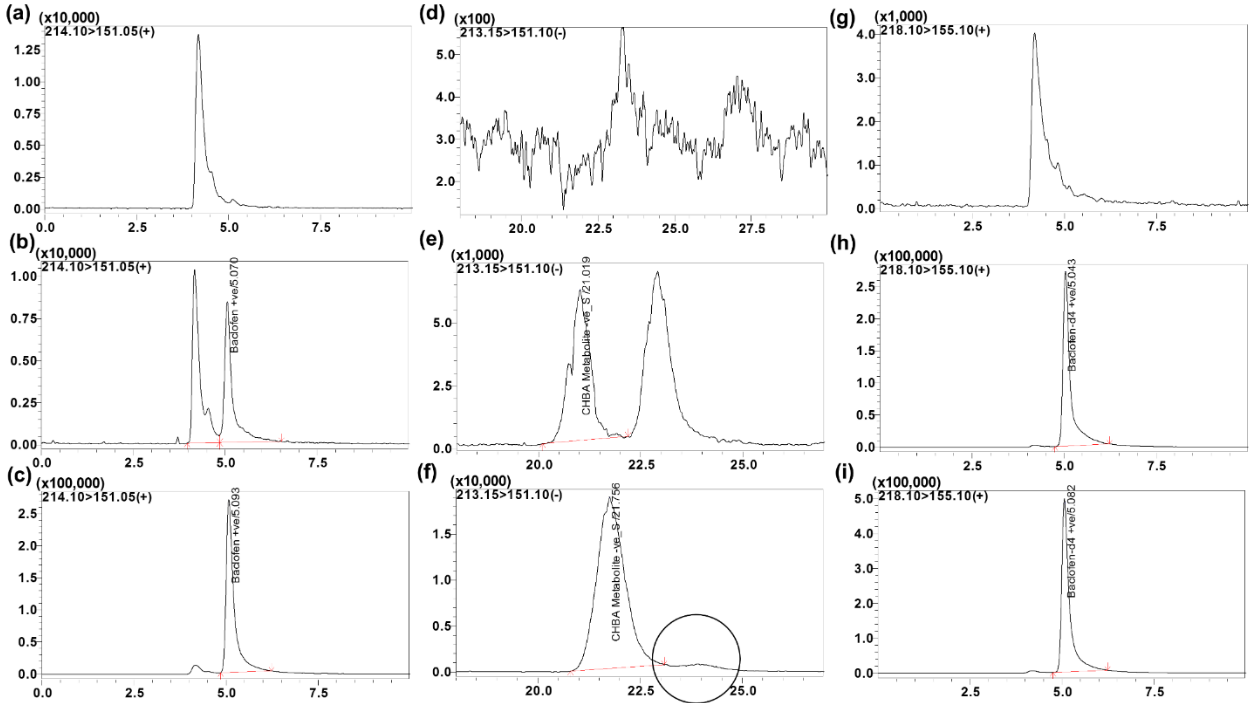

Supplement: Supplementary file 1 [file molecules-25-00250-s001.pdf]
